# Microstructures and Mechanical Properties of Commercially Pure Ti Processed by Rotationally Accelerated Shot Peening

**DOI:** 10.3390/ma11030366

**Published:** 2018-03-02

**Authors:** Zhaowen Huang, Yang Cao, Jinfeng Nie, Hao Zhou, Yusheng Li

**Affiliations:** Nano and Heterogeneous Structural Materials Center, School of Materials Science and Engineering, Nanjing University of Science and Technology, Nanjing 210094, China; Ryan_ZWHuang@163.com (Z.H.); y.cao@njust.edu.cn (Y.C.); niejinfeng@njust.edu.cn (J.N.)

**Keywords:** rotationally accelerated shot peening, titanium, gradient structure, deformation mechanism, mechanical properties

## Abstract

Gradient structured materials possess good combinations of strength and ductility, rendering the materials attractive in industrial applications. In this research, a surface nanocrystallization (SNC) technique, rotationally accelerated shot peening (RASP), was employed to produce a gradient nanostructured pure Ti with a deformation layer that had a thickness of 2000 μm, which is thicker than those processed by conventional SNC techniques. It is possible to fabricate a gradient structured Ti workpiece without delamination. Moreover, based on the microstructural features, the microstructure of the processed sample can be classified into three regions, from the center to the surface of the RASP-processed sample: (1) a twinning-dominated core region; (2) a “twin intersection”-dominated twin transition region; and (3) the nanostructured region, featuring nanograins. A microhardness gradient was detected from the RASP-processed Ti. The surface hardness was more than twice that of the annealed Ti sample. The RASP-processed Ti sample exhibited a good combination of yield strength and uniform elongation, which may be attributed to the high density of deformation twins and a strong back stress effect.

## 1. Introduction

Titanium is attractive in the biomedical engineering, aerospace and automobile industries, due to its excellent biocompatibility, high specific strength, and novel chemical corrosion resistance [1,2,3]. However, coarse-grained Ti has a relatively low strength and low hardness, which restricts the wide application of Ti as a promising biomaterial material [4,5]. Severe plastic deformation (SPD) techniques, such as accumulative roll bonding (ARB) [6,7], equal channel angular press (ECAP) [8,9], and high-pressure torsion (HPT) [10,11] can significantly enhance the strength of metallic materials through their high capability of grain refinement [12,13,14]. However, these processes are either inadequate for producing big-sized samples, or time-consuming. Moreover, high strength bulk nanocrystalline (nc) materials produced by SPD techniques usually exhibit poor ductility due to the lack of strain-hardening ability, and fail catastrophically under tensile deformation tests [12,15].

Surface nanocrystallization (SNC), which was proposed by Lu et al., is a surface SPD technology for processing gradient structured materials [16,17]. It has been reported that a gradient structure with nanograins on the surface can bring a better strength-ductility combination to many materials [18,19,20,21]. Wen et al. [22] found that both the yield strength ($\sigma_{y}$) and ultimate tensile strength ($\sigma_{\mathrm{uts}}$) of gradient structured Ti increased by ~100 MPa after surface mechanical attrition treatment (SMAT) processing respectively, while retaining ~59% of the uniform elongation of the coarse-grained (CG) counterpart. Based on our current and still expanding knowledge, the outstanding mechanical properties of gradient nanostructured materials is mainly attributed to the unique inhomogeneous structure with CGs in the core and nanograins on the surface layer, which induces strong back stress strengthening to the material [23,24]. The very high surface hardness and good strength–ductility synergy endow the gradient structured materials with high application potential [19,25]. However, due to the restrictions of the SMAT technique, the most common sample thickness prepared by SMAT processing is only ~1 mm [20]. For industrial applications, it is a challenge to develop new techniques of producing gradient structured materials with thicker deformation zones [25]. 

More recently, a novel SNC technique, rotationally accelerated shot peening (RASP), was developed [26]. The RASP technology, which increases the momentum of steel balls with centrifugal acceleration, can produce a higher impact energy than conventional SNC processes, e.g., SMAT and conventional shot peening technique [27]. As a result, the RASP process can produce large deformation zones with a high grain refinement efficiency. For example, even the center of a 4-mm thick 5052 Al alloy was plastically deformed after RASP processing [28]. Most importantly, RASP technology can be easily scaled up for industrial applications. The purpose of this paper is to reveal the microstructures and mechanical properties of a Ti sample processed by the RASP technology, and make the fundamental knowledge of the gradient nanostructured Ti readily available for possible medical and industrial applications. 

## 2. Experimental

The materials used in this work are commercially pure titanium (grand TA2) plates with the dimensions of 100 mm × 60 mm × 4 mm. The chemical composition (wt %) is: O 0.15%, N 0.01%, C 0.01%, Fe 0.03%, and balanced Ti. Before RASP treatment, in order to obtain a uniform structure with equiaxial grains, Ti samples were annealed at 750 °C for 2 h with a nitrogen protective atmosphere, followed by a furnace cooling to room temperature. The annealed Ti plates were polished with silicon carbide paper in order to remove oxides and contaminations from the surface. The details of the RASP setup and processing have been described previously [26]. In brief, 2-mm diameter steel balls were accelerated to a high speed by the centrifugal force and impact with the Ti sample surface. RASP treatments were conducted on the Ti sample at a velocity of 20 m/s for a total duration of 30 min. During the RASP process, the Ti sample was rotated at a speed of 15 rpm, and both sides of the samples were processed. In order to avoid temperature increase on the surface, the RASP process was stopped every five minutes to let the sample cool to room temperature. 

An optical microscope (OM; Axio Vert A1, ZEISS, Oberkochen, Germany) was used to observe the gradient structures of the RASP-processed Ti. Microstructures of the samples were characterized in detail by means of electron backscattering diffraction (EBSD) analysis, which was performed on a scanning electron microscope (SEM, Quant 250 FEG, FEI, Hillsboro, OR, USA), operating at 20-kV applied voltage and with a scanning step size of 0.8 μm. For OM and EBSD investigations, the samples were prepared by mechanical grinding and electropolishing in order to obtain a mirror surface. The solution that was used in electropolishing process was a mixture of perchloric acid and acetic acid with a volume ratio of 1:9. Etching before OM observation is carried out in a solution of 20% hydrofluoric acid, 20% nitric acid, and 60% deionized water. High magnification investigation was done by using transmission electron microscopy (TEM, Tecnai G2 20, FEI, Hillsboro, OR, USA) operating at 200 kV of applied voltage. TEM were conducted to examine the microstructures from the surface to the interior of the Ti samples. 

Microhardness through the depth of the RASP-processed Ti and the as-received Ti were measured by using a microhardness tester (HMV-G 21DT, Shimadzu, Tokyo, Japan) with a load of 100 g and a holding time of 15 s. Each hardness data point was an average from 10 indents with a corresponding error-bar. Uniaxial tensile tests were carried out on a tensile tester (LFM-20, Walter +Bai AG, Löhningen, Switzerland), with a strain rate of ~3 × 10^−3^/s at room temperature, and all of the tests were performed at least three times.

## 3. Results and Discussion

**Figure 1:**
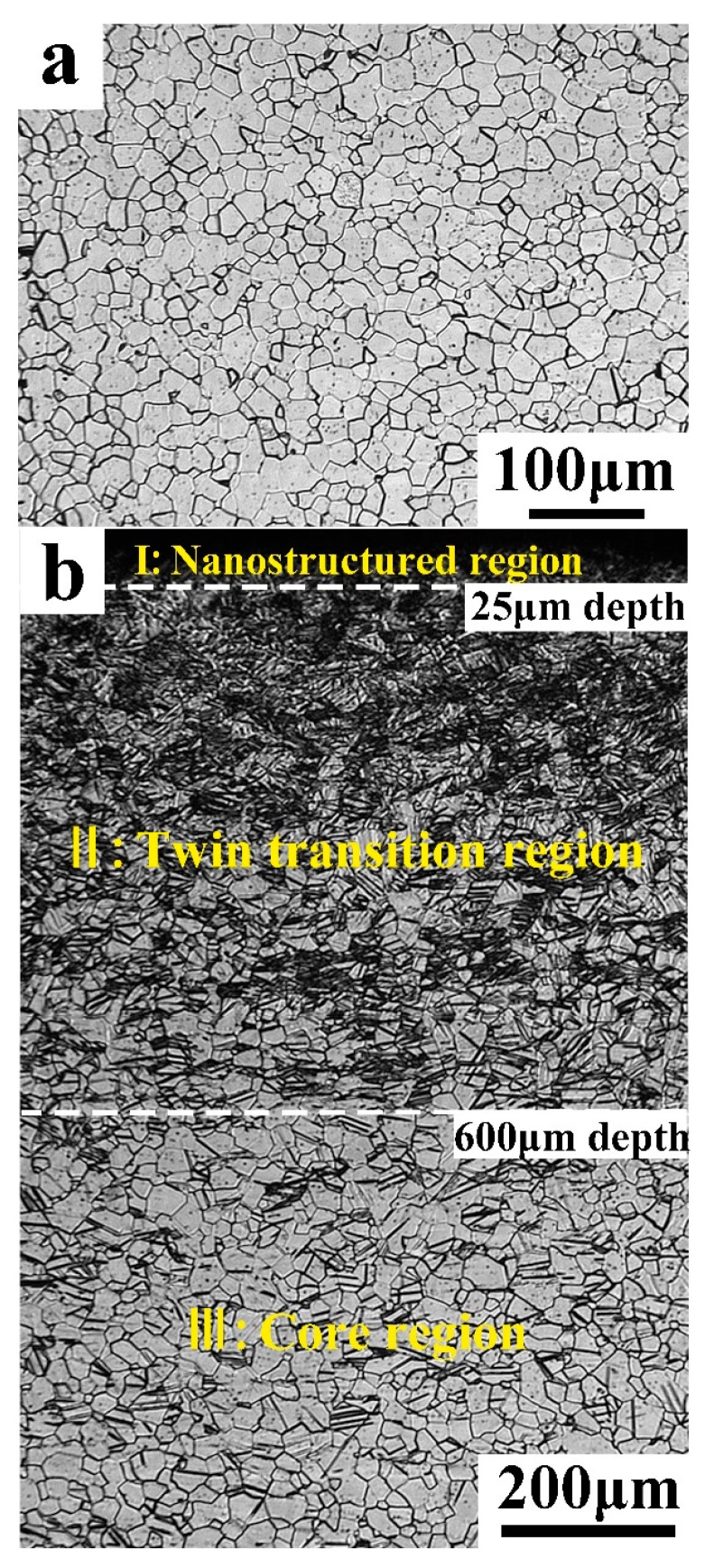
Optical micrographs of: (**a**) the as-annealed Ti, and (**b**) the rotationally accelerated shot peening (RASP)-processed Ti.

Figure 1a shows the microstructure of the as-received Ti, which consists of equiaxed grains, with an average grain size of ~18 μm. A few twins can be observed in the Ti after annealing. After the RASP treatment, a gradient structure without delamination is introduced, as shown in Figure 1b. Obviously, the whole sample has been deformed, but to a different extent through the depth. The deformed layer is about 2000 μm thick, from the surface all the way to the center, i.e., the whole specimen is deformed, and no original zone is left. Therefore, a deformation layer across the whole thickness of the sample is fabricated. The thick deformation layer was attributed to the high energy of RASP processing, compared to that of SMAT and conventional shot peening [26]. The processing speed is higher than that of SMAT, while the shot ball size is bigger than that of shot peening. Furthermore, the deformation layer can be divided into three regions: a core region in the center, a twin transition region in halfway, and a nanostructured region close to the surface. The core region consists of large grains with low densities of dislocations [29,30,31,32]. The twin transition region is the middle layer between the core region and the nanostructured region, which is located at the depth range from ~25 µm to 600 µm. Coarse grains coexist with a large amount of multisystems twins in this region. Twins with different orientations incise each other, forming a “twinning intersection”-shaped microstructure to refine the grain size. The nanostructured region is the top layer, with a thickness of ~25 μm. The nanostructured region contains very fine grains. The microstructure at this region is hardly resolved by the optical microscope and the EBSD observation.

**Figure 2:**
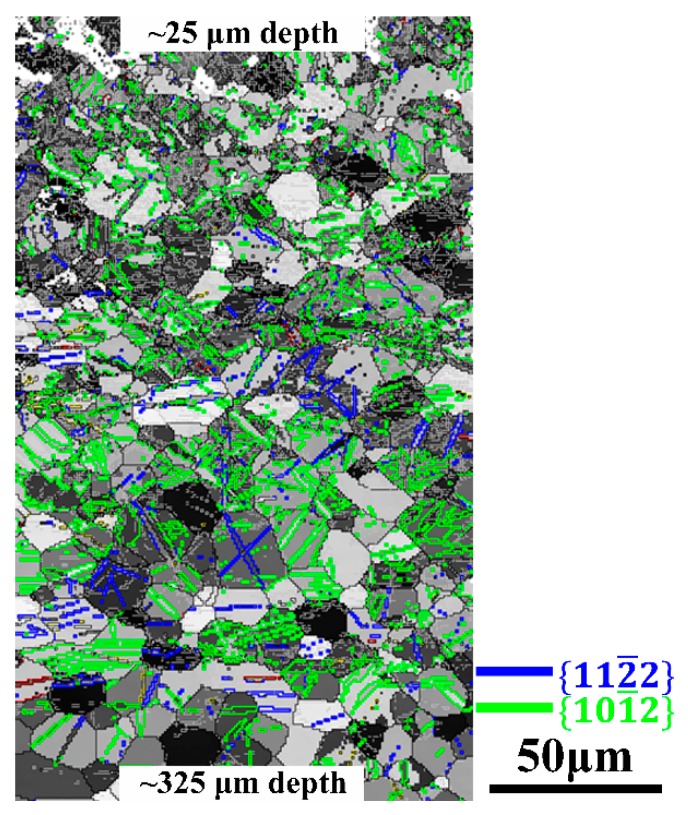
An electron backscattering diffraction (EBSD) image at the depth of 25 μm to 325 μm from rotationally accelerated shot peening (RASP)-processed Ti; boundaries are colored black, white, blue and green corresponding to high-angle grain boundaries (HABs), low-angle grain boundaries (LABs), $\left\{11\bar{2}2\right\}$ compression twins (CTs) and $\left\{10\bar{1}2\right\}$ extrusion twins (ETs), respectively.

In order to understand the gradient structures, especially the twin transition region, EBSD observations at the depth range from ~25 μm to 325 μm of RASP-processed Ti are shown in Figure 2. The boundaries that are colored black, grey, blue and green are high-angle grain boundaries (HABs), low-angle grain boundaries (LABs), $\left\{11\bar{2}2\right\}$ compression twin (CT) boundaries, and $\left\{10\bar{1}2\right\}$ extrusion twin (ET) boundaries, respectively [33]. Obviously, ETs are the major type of twinning in the sample. For the ETs and CTs in Ti, the twinning shear can be estimated as [34]:

(1) $$S_{\left\{10\bar{1}2\right\}}=\left(\gamma^{2}-3\right)/3^{\frac{1}{2}}\gamma$$

(2) $$S_{\left\{11\bar{2}2\right\}}=2\left(\gamma^{2}-2\right)/3\gamma$$

where γ is the c/a ratio of the HCP metal, and it is 1.587 for Ti. By using these formulae, the twinning shear of ETs and CTs of Ti are calculated as 0.175 and 0.218, respectively. The lower twinning shear of ETs means that less stress/energy is needed to activate the twinning system, which makes ETs the dominant twinning type in RASP-processed Ti. Besides, previous investigations have revealed that the stress concentration at grain boundaries induced by $\left\{10\bar{1}2\right\}$ ETs can be accommodated by a prism slip in neighboring grains [35]. It can reduce the probability of crack generation in Ti during RASP treatment, which may attribute to a better ductility of RASP-processed Ti. Moreover, the cogeneration of $\left\{11\bar{2}2\right\}$ CTs and $\left\{10\bar{1}2\right\}$ ETs is believed to significantly benefit the strain hardening of the material and prevent delamination to a certain extent [36]. 

**Figure 3:**
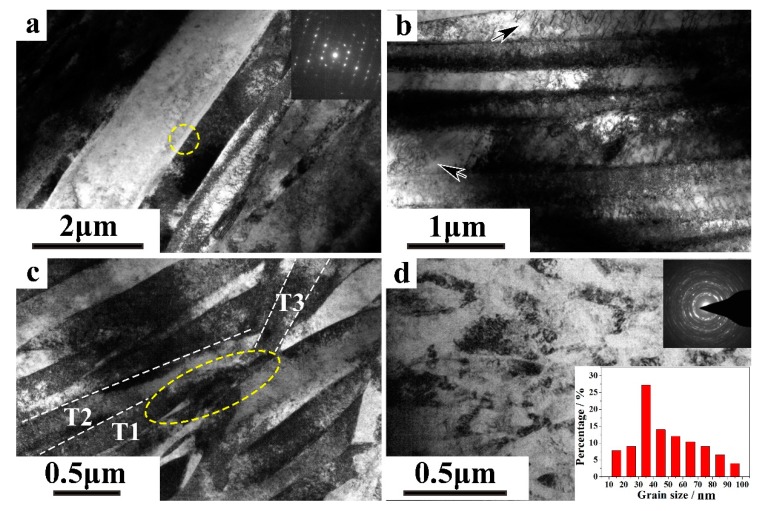
TEM images of representative microstructures at different regions through the depth of the RASP-processed Ti: (**a**,**b**) monosystem twinning and high density dislocation structures in the core region, (**c**) multisystems twinning in the twin transition region, and (**d**) nano-/ultrafine grains in the nanostructured region, corresponding grain size distribution reveals that an average grain size of ~50 nm is fabricated in Ti.

Figure 3 gives the TEM images of typical microstructures at different regions through the depth of the RASP-processed Ti. As shown in Figure 3a, b, high densities of twins and dislocations are found in the core region. Monosystem twinning is activated, and parallel twin lamellas cut the initial coarse grains into segments. Meanwhile, dislocation activities are pronounced inside both twins and parent grains to accommodate plastic deformation as shown in Figure 3b. Dislocation walls formed by dislocations pile up, and accumulation is marked by black arrays, which are probably the sources of LABs in the core region [29,37]. As mentioned above, the twin transition region is the layer with a larger deformation strain than that of core region. The resolved shear stress (RSS) of other twinning systems in a twin transition region increases significantly. Figure 3c shows that multisystems twinning is activated in the twin transition region, and they interact with each other with different orientations. The parallel twins, T1 and T2, are cut by T3, forming a region of smash and stagger of twin depicted by the yellow dash circle. Under the combination effect of slip and twinning, grains are refined into a smaller size that twinning critical resolved shear stress (CRSS) is hard to reach. Dislocation slip becomes dominant in plastic deformation accommodation. Figure 3d shows the microstructure in the nanostructured region, which exhibits uniform distribution of grains with random orientations. Grains are irregular, and do not have well-defined boundaries due to a high dislocation density and large internal stress [38]. The corresponding grain size distribution is derived from several TEM images, and hundreds of grains are taken into consideration. The result shows that grains are refined into a nanoscale with an average grain size of ~50 nm, while those with a size scale range from 30 nm to 40 nm take up a large portion, ~27%. It indicates that nanograins are formed in Ti by RASP treatment.

**Figure 4:**
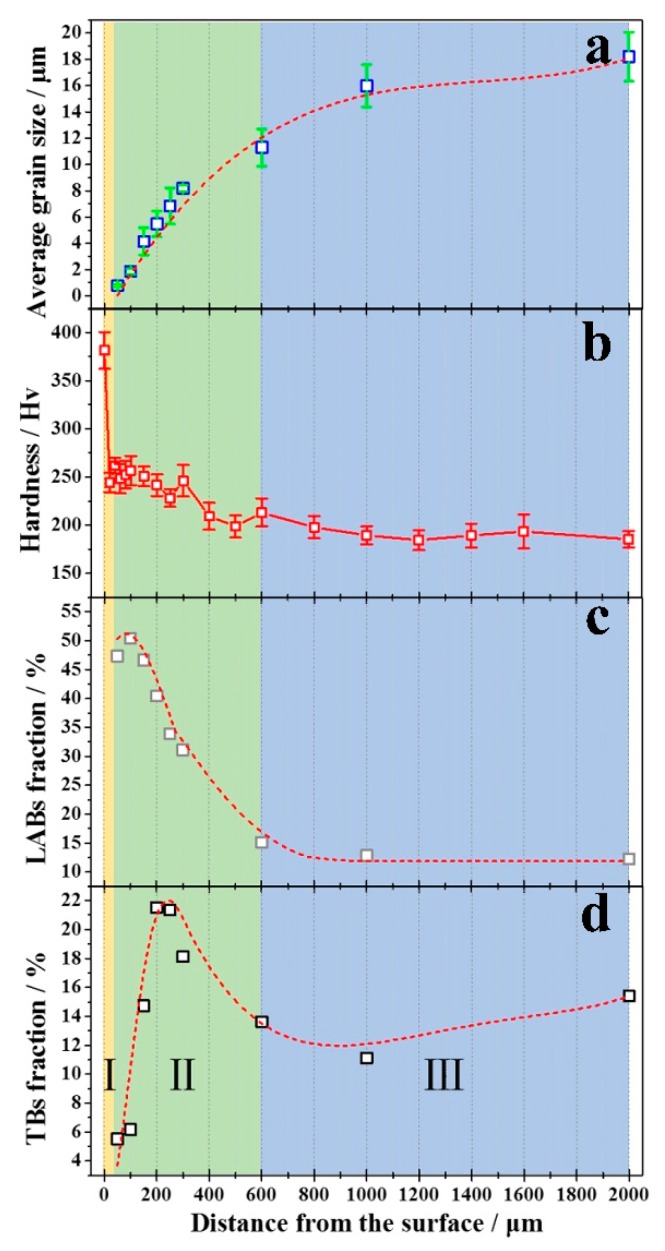
Distribution of (**a**) grain size; (**b**) hardness; (**c**) fractions of LABs and (**d**) fractions of twin boundaries (TBs) from the surface to the interior of the RASP-processed Ti.

Statistical results obtained by using EBSD technique (Figure 4a,c,d) and the hardness test (Figure 4b) are given. EBSD analysis has been done at the depth from 2000 μm to 50 μm of RASP-processed Ti. Figure 4a shows the grain size distribution of RASP-processed Ti. Grains with boundary misorientations larger than 15° are considered to be individual in the calculation, as well as twin boundaries. Besides, the length of the short axis is regarded as the grain size. The grain size decreases monotonously with the decreasing distance to the surface. According to the Hall-Petch relationship, grain refinement can enhance the hardness of materials, owing to grain boundary strengthening. The hardness of RASP-processed Ti on the surface is 381 HV, which is much higher than the 178 HV of annealed CG Ti. Meanwhile, the hardness decreases gradually as the depth from the surface increases, as shown in Figure 4b. In the core region of RASP-processed Ti (region III, colored by blue), the hardness remains higher than that of annealed Ti due to the residual stress and substructure, which is similar to the RASP-processed 5052 Al alloy [28]. When approaching to the surface, twin density increases with increasing deformation strain, and leads to an obvious enhancement of hardness in the twin transition region (region II, colored by green). While on the surface (nanostructures region, region I, colored by yellow) of RASP-processed Ti, the hardness reaches the maximum value attributed to the nanograins and abundant substructure. 

Figure 4c shows the distribution of the LABs fraction of RASP-processed Ti through the depth. The LABs fraction is ~12% at the depth of 2000 μm, then increases monotonously to about 50% at the depth of 100 μm, and finally decreases to 47% at a depth of 50 μm. The fraction of LABs is correlated to the dislocation density, and the high fraction of LABs at the surface layer of the sample indicates a high dislocation density, which is attributed to high strain and high strain rate at the local area. The reason of the turning point at 100-μm depth can be attributed to the transformation of LABs to HABs at the stage, since dislocation accumulation tends to be saturated, and a higher deformation strain in the top surface triggers the transformation [29,31,37]. 

Figure 4d shows the fraction of twin boundaries (TBs) in RASP-processed Ti, which increases generally from the center to the surface, and a peak with a maximum fraction of ~22% appears at the depth of 200 μm. With the decreasing distance from the surface, twinning is stimulated to accommodate the strain. The microstructure changed from monosystem twins to multisystems twins with increasing deformation strain, and finally led to a twinning intersection in parent grains as shown in Figure 3c, which results in the increase of the TBs fraction. Notice that there are two major influencing factors of twin boundary density in metallic metals, i.e., deformation strain and grain size [39,40,41]. Usually, reducing grain size results in higher twinning stress in the fine grain region, which impedes twin generation [42,43,44]. This would be an explanation for the decrease of the TBs fraction in the region with a depth of less than 200 μm to the surface.

**Figure 5:**
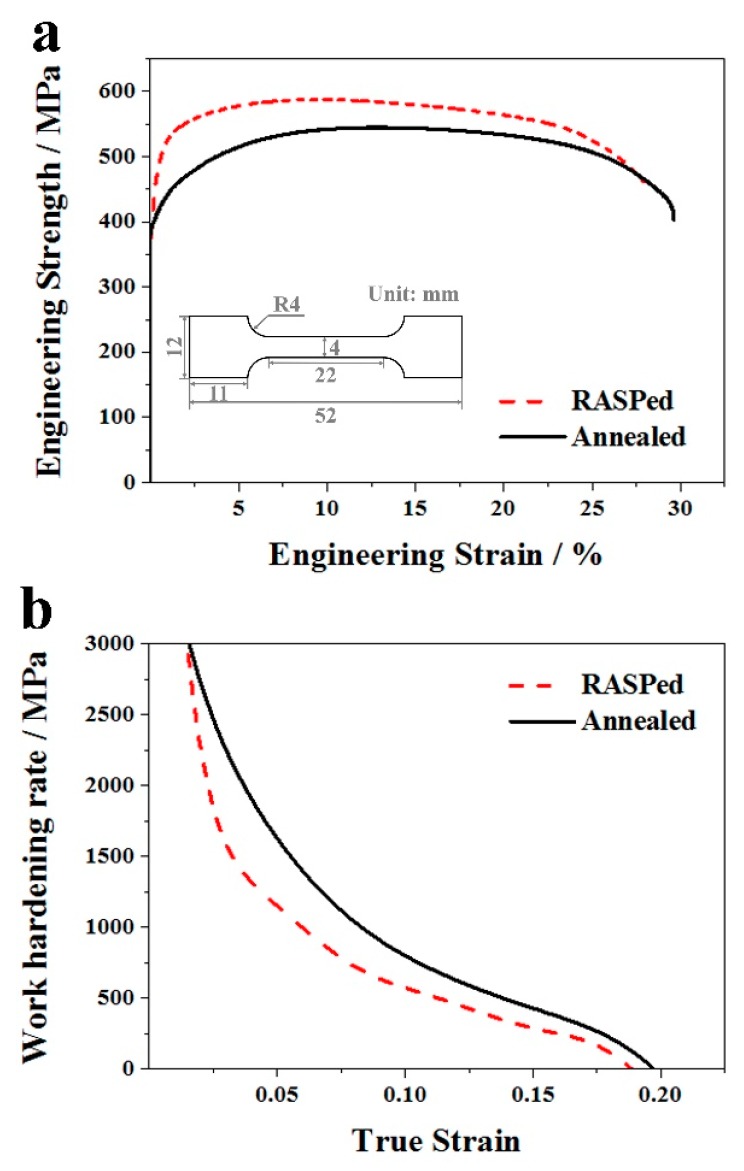
(**a**) Engineering stress-strain curves, and the size of the “dog bone” shape tensile sample is shown, (**b**) strain hardening rate (θ = dσ/dε) versus the true strain of as-annealed Ti and RASP-processed Ti.

Figure 5a shows the tensile curves of annealed and RASP-processed Ti samples. The dimensions of the samples for the tensile test are given as an inset. RASP-treated Ti exhibits an obvious strength enhancement compared with the annealed Ti. The σ_y_ and σ_uts_ of Ti increase from 378 MPa and 551 MPa in CG Ti to 535 MPa and 593 MPa in RASP-processed Ti, respectively. A microstructural analysis indicated that the significant improvement of σ_y_ was related to the increase of dislocation density, and the Hall-Petch strength resulted from grain refinement [45,46]. A slight decrease of uniform elongation (ε_u_) from 12.5% in CG Ti to 10.5% in RASP-processed Ti is realized after RASP treatment, while the RASP-processed Ti was still deemed to possess a good combination of strength and ductility. Figure 5b gives the strain-hardening rate (θ = dσ/dε) versus the true strain of CG and RASP-processed Ti samples. Large grains can provide ample space for dislocation movement, which contributes to the excellent working hardening ability of CG Ti. After RASP treatment, high density of deformation twins and dislocation pile-ups are found in Ti (Figure 3b,c), resulting in higher strength and inevitable lower ductility. Nevertheless, work-hardening lines for RASP-processed Ti and annealed Ti are nearly parallel during the plastic straining process, showing that RASP-processed Ti has a good work-hardening ability. 

The excellent mechanical properties of RASP-processed Ti may be attributed to two reasons. (1) The first is a high density of deformation twins. Similar to conventional high-angle grain boundaries, twins can act as dislocation slip barrier, and result in a significant increase of σ_y_. Moreover, dislocations travel parallel to the twin boundaries, and twins provide ample space for dislocation movement, which make dislocation slip easier, and thus reserve admirable work hardening ability/ductility [37,47,48,49]. (2) The second reason for the excellent mechanical properties of RASP-processed Ti is back stress strengthening. The gradient structure can be generally regarded as the integration of many layers with different grain size and hardness (Figure 4a,b); therefore, a high density of soft/hard interfaces exists in the gradient structured RASP Ti sample. During plastic straining, strain gradients and plastic incompatibilities between neighboring layers lead to obvious back stress strengthening and work hardening, which benefit both the strength and ductility [23,24]. It is likely that the back stress played a significant role in producing the superior mechanical properties in the gradient Ti, similar to that in IF steel [20].

**Figure 6:**
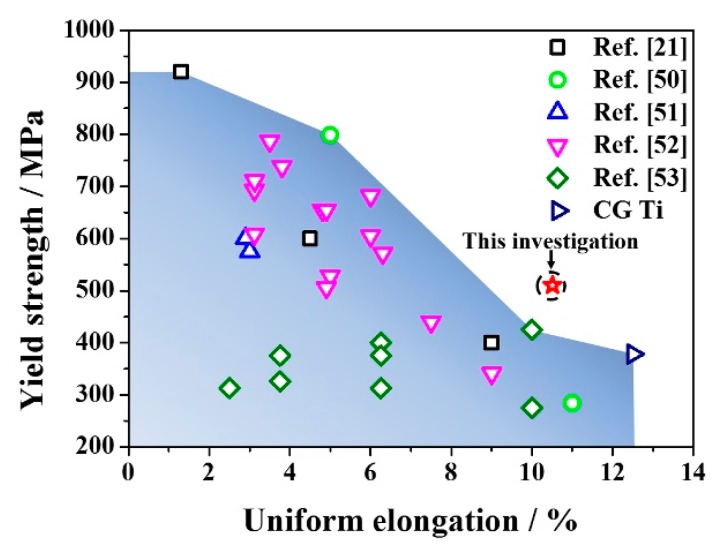
A plot of yield strength and uniform elongation for TA2 in present and other work from literatures [21,50,51,52,53].

Figure 6 summarizes a set of tensile property data of TA2 processed by different SPD techniques, e.g., SMAT [22], ARB [50], ECAP [51], ECAP + extrusion [52], and laser shock peening [53]. The σ_y_-ε_u_ data of the present RASP-processed Ti was also plotted in the figure. The majority of the data points are within a shadow area, implying a trade-off between strength and ductility. Our work can be distinguished, since the RASP-processed Ti data is out the shadow scope. More importantly, the RASP technique is flexible in adjusting processing parameters such as ball size, ball speed, processing time, sample thickness, etc.; thereby, more tensile property data are expected and the relevant research is ongoing. The good combination of high surface hardness (Figure 4b) and strength-ductility synergy in RASP-processed Ti (Figure 5 and Figure 6) is promising for biomedical applications [2,3].

## 4. Conclusions

(1) A gradient structure was introduced to a Ti sample by RASP treatment. The processed sample had a deformed layer of 2000 μm in depth without obvious delamination. The averaged grain size of the RASP-processed Ti decreased from ~18 μm in the center to 50 nm in the surface.
(2) Different deformation mechanisms were operative during the RASP processing. Deformation twinning was dominant in the core region. The twin volume fraction increased with the decreasing of depth. Meanwhile, dislocation slip occurred inside both twins and parent grains. The LABs fraction increased gradually due to dislocation pile-up and accumulation. Multisystem twinning is activated in the twin transition region. Twin interaction led to further grain refinement, and a smaller grain size resulted in an increase of twinning stress. Hence, twinning is difficult to activate in the nanostructured region with very fine grains, and dislocation activities are dominant in the region.
(3) Hardness gradient was observed in the RASP-processed Ti through the depth. The hardness in the top surface (381 HV) is more than twice that of its CG counterpart (178 HV).
(4) The RASP-processed Ti showed significant strengthening; the σ_y_ and σ_UTS_ increase from 378 MPa and 551 MPa to 535 MPa and 593 MPa, respectively. The uniform elongation of the RASP-processed Ti showed a slight decrease from 12.5% to 10.5%. The excellent strength-ductility combination was attributed to the high density of deformation twins, and the back stress strengthening and work hardening.
